# Molecular Epidemiology of Carbapenem-Resistant *Acinetobacter baumannii* Isolated from War-Injured Patients from the Eastern Ukraine

**DOI:** 10.3390/antibiotics9090579

**Published:** 2020-09-05

**Authors:** Paul G. Higgins, Ralf Matthias Hagen, Andreas Podbielski, Hagen Frickmann, Philipp Warnke

**Affiliations:** 1 Institute for Medical Microbiology, Immunology, and Hygiene, University of Cologne, 50935 Cologne, Germany; paul.higgins@uni-koeln.de; 2 German Centre for Infection Research (DZIF), partner site Bonn-Cologne, 50935 Cologne, Germany; 3 Department of Microbiology and Hospital Hygiene, Bundeswehr Central Hospital Koblenz, 56070 Koblenz, Germany; ralfmatthiashagen@bundeswehr.org; 4 Institute for Medical Microbiology, Virology and Hygiene, University Medicine Rostock, 18057 Rostock, Germany; frickmann@bnitm.de (H.F.); philipp.warnke@med.uni-rostock.de (P.W.); 5 Department of Microbiology and Hospital Hygiene, Bundeswehr Hospital Hamburg, 20359 Hamburg, Germany

**Keywords:** *Acinetobacter baumannii* complex, war injury, Ukraine, epidemiology, sequence typing, carbapenem resistance, resistance gene, crisis zone

## Abstract

Recently, a total of 32 carbapenem- and fluoroquinolone-resistant *Acinetobacter baumannii* (Ab) isolates was isolated from war-injured patients who were treated at German Bundeswehr Hospitals, and preliminarily typed by “DiversiLab” repetitive elements sequence-based (rep-) PCR. Core genome-based sequence typing was also used to provide more detailed epidemiological information. From the clusters observed by rep-PCR, selected Ab strains were subjected to Next Generation Sequencing (NGS) in order to compare them with international outbreak-associated Ab strains and to identify MLST (multi-locus sequence type) lineages, as well as to identify known resistance genes. Accordingly, NGS indicated higher diversity than rep-PCR, but also confirmed likely transmission events. The identified acquired carbapenem-resistant genes comprised *bla*_OXA-23_, *bla*_OXA-72_ and *bla*_GES-12_, as well as various other intrinsic and acquired resistance-associated genetic elements. All isolates clustered with the previously identified international clonal lineages IC1, IC2, IC6 and IC7, with corresponding Pasteur sequence types ST1, ST2, ST78 and ST25, respectively. In conclusion, the assessment confirmed a broad spectrum of resistance-associated genes in Ab isolated from war-injured patients from the Eastern Ukraine, and provided the first insights into locally abundant clonal lineages.

## 1. Introduction

More than a decade ago, drug resistant or even multi-drug resistant *Acinetobacter baumannii* were described as an issue of concern in patients with war injuries [1,2,3,4], and remain a topic of ongoing relevance [5,6,7]. Typically, nosocomial transmission is more likely in patients with combat wounds than is auto-infection, due to previous colonization with *A. baumannii* [8,9,10,11,12] with little or no effects of point-of-injury application of antimicrobial drugs [13]. The majority of reports on warfare-associated *A. baumannii* infections in the international literature are focused on the recent military conflicts in Iraq [14,15,16,17,18,19] and Afghanistan [20,21,22,23,24,25,26].

From the Eastern Ukrainian conflict, infections of war-injured patients with multi-drug resistant *Acinetobacter* spp. have been described [27]; however, a detailed investigation into the species distribution is lacking. *Acinetobacter* spp. were particularly abundant as part of the wound flora in war-injuries in later stages of the wound consolidation process, while Gram-positive bacteria dominated at the beginning [27]. Carbapenem-resistance in *A. baumannii* bloodstream isolates from Ukrainian hospitals is high, with a prevalence of 63.2% reported in a multi-center study of Ukrainian hospitals between 2013 and 2015 [28]. A recent study from 2019 showed that 78.6% of the *A. baumannii* isolates causing healthcare associated infections in a Ukrainian intensive care unit were carbapenem-resistant [29]. As this is a challenge for antimicrobial therapy, physicians from the Ukraine have even reported desperate therapeutic attempts, comprising doxycline, in cases of aminoglycoside-resistant *A. baumannii* [30].

The available data on underlying carbapenem resistance mechanisms in the Ukraine are even more scare, and mostly focus on *Enterobacteriaceae* rather than non-fermentative rod-shaped bacteria such as *A. baumannii* [31,32,33].

As recently described in detail [34], 32 carbapenem- and fluoroquinolone-resistant *A. baumannii* isolates, as identified by biochemistry and matrix-assisted laser-desorption-ionization time-of-flight mass spectrometry (MALDI-TOF-MS), were isolated from 21 male patients with war-injuries from the Eastern Ukrainian conflict, who were treated at Bundeswehr Hospitals in 2014 and 2015 for humanitarian reasons. As indicated by preliminary typing, applying repetitive elements sequence-based (rep-) PCR (DiversiLab, BioMérieux, Marcy l’étoile, France), the colonization or infection of patients with more than one clone indicated high colonization pressure. As such, only four obvious clonal clusters next to rare singletons were observed [34], suggesting quite limited clonal diversity.

In the course of the same assessment [34], an attempt at the identification of carbapenemase genes was started, based on three multiplex PCRs targeting 11 carbapenemase genes, which had been originally designed for carbapenem-resistant *Enterobacteriaceae* [35]. As the resistance mechanisms typically abundant in *A. baumannii* were not covered by this approach, and known resistance mechanisms against fluoroquinolones in *A. baumannii* were not even addressed [36], the approach failed to identify the genetic background of the phenotypically observed resistance of the isolates.

To resolve this information gap, eight isolates were selected, based on their unique rep-PCR patterns from a previous study [34], for whole genome sequencing, and were analyzed by core genome multi locus sequence typing (cgMLST) [37]. The aim of this assessment was to provide additional epidemiological information on the international distribution of resistant bacterial clones, and on detectable molecular resistance mechanisms.

## 2. Results

### 2.1. Core Genome-Based Confirmation on Species Level and Clustering with International Outbreak Strains

**Figure 1:**
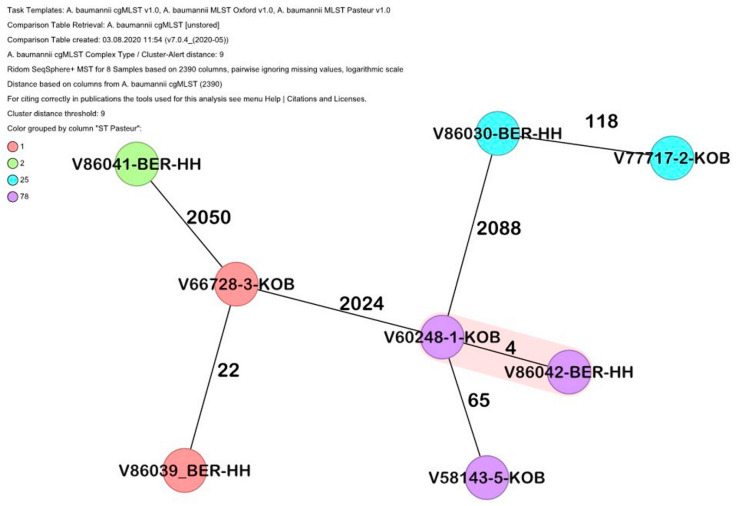
Minimum spanning tree of the *A. baumannii* based on 2390 target alleles (core genome). Isolate numbers are within the nodes, and the numbers between the nodes indicate the number of alleles that were different. Isolates are colored based on their Pasteur sequence type.

All isolates were confirmed as *A. baumannii* by the *gyrB* multiplex PCR, and this was confirmed by their genome sequences. cgMLST analysis revealed that the isolates clustered with the previously identified international clonal lineages IC1, IC2, IC6 and IC7 [38], as shown in Figure 1.

Of note, a difference of only four alleles was recorded for strain V60248-1-KOB, isolated from a patient at the Bundeswehr Central Hospital in Koblenz, Germany, and V86042-BER-HH, isolated in the laboratory of the Bundeswehr Hospital, Berlin, from a patient transferred to the north of Germany, suggesting a common source of infection.

The isolates were assigned sequence types based on the two 7-loci multi-locus sequence typing (MLST) schemes, the recently comparatively assessed Oxford scheme and Pasteur schemes [39], which identified the Oxford sequence types ST231 (n = 2), ST440 (n = 1), ST690 (n = 1), ST944 (n = 2), ST1102 (n = 1) and ST2144 (n = 1), as well as the Pasteur sequence types ST1 (n = 2), ST2 (n = 1), ST25 (n = 2) and ST78 (n = 3), respectively (Table 1).

### 2.2. Identified Molecular Resistance Mechanisms

Analysis of antimicrobial resistance determinants, ordered by strain, MLST type and international clonal lineage, are summarized in Table 1.

Beta-lactamase, sulfonamide and aminoglycoside resistance genes were the most abundant in the isolates, while fluoroquinolone resistance was associated with the double substitution Ser83-Leu and Ser80-Leu in GyrA and ParC, respectively. Two isolates were in possession of the fluoroquinolone/aminoglycoside modifying enzyme aac(6′)Ib-cr-like. Five variants of the intrinsic *bla*_ADC-25-like_ were detected in the isolates, with the variants associated with their clustering by cgMLST. Furthermore, the intrinsic *bla*_OXA-51-like_ variants *bla*_OXA-64_, *bla*_OXA-66_, *bla*_OXA-69_ and *bla*_OXA-117_, respectively, were associated with particular clonal lineages as confirmed by Oxford and Pasteur MLST, as well as cgMLST (Figure 1, Table 1). All carbapenem-resistant isolates had an acquired carbapenemase gene, comprising *bla*_OXA-23_ or *bla*_OXA-72_ (*bla*_OXA-40-like_), while the elevated minimum inhibitory concentrations of carbapenems in isolate V667283-KOB were most likely caused by *bla*_GES-12_. Carbapenem-susceptible isolate V77717-2-KOB possessed only the intrinsic *bla*_OXA-64_. No isolates had IS*Aba1* associated with their intrinsic *bla*_OXA-51-like_.

## 3. Discussion

This study was performed to allow a more detailed look at the micro-epidemiology of carbapenem- and fluoroquinolone-resistant *A. baumannii* isolates from patients with war-injuries as consequences of the military conflict in the Eastern Ukraine, who were treated at German Bundeswehr Hospitals in 2014 and 2015. Indeed, cgMLST- and MLST-based typing applying the Pasteur and Oxford schemes [39] provided more details than preliminary rep-PCR-typing, based on which clustering the sequenced strains were chosen [34]. Due to the economic constraints of this investigator-initiated assessment without external funding, the analyses were restricted to eight isolates, i.e., one isolate from each rep-PCR-cluster and the four singletons with distinct patterns in rep-PCR [34]. It is likely that the inclusion of all 32 strains might have allowed a slightly more differentiated view by revealing differences beyond the discriminatory potential of rep-PCR, an admitted limitation of this study. Of note, all assessed isolates were associated with the previously described international outbreak clones [38].

The use of cgMLST-typing did not only show differences, but also striking similarities. Based on the rep-PCR profiles, the clustering suggested the clonal identity of isolates collected from hospitalized patients at different Bundeswehr Hospitals, suggesting transmission events during the evacuation flights from the Ukraine, or during treatment in medical facilities in the patients’ home country [34]. This hypothesis was confirmed by the presence of only four allelic differences in cgMLST between two *A. baumannii* isolates from the laboratories of the Bundeswehr Central Hospital Koblenz and the Bundeswehr Hospital Berlin (V60248-1-KOB and V86042-BER-HH, Figure 1); as such, nosocomial transmission in the same Bundeswehr Hospital was excluded. At the same time, rep-PCR suggested a link between V66728-3-KOB and V77717-2-KOB, but this link was disproven with the cgMLST data. Furthermore, rep-PCR failed to show a link between isolates V86039-BER-HH and V66728-3-KOB, which had 22 alleles different, thus demonstrating the higher resolution of genome-based typing [40].

Focusing on the resistance genes, the carbapenem-resistant isolates were all in possession of either *bla*_OXA-23_ or *bla*_OXA-72_, which are the most common carbapenem resistance determinants in *A. baumannii*, especially in Europe [41]. Despite the low number of allelic differences between some isolates, there were significant differences in their resistome.

Scarce data exist for *A. baumannii* from Ukraine; however, recently three isolates were investigated in Germany from patients injured in Russia and repatriated. These isolates all had *bla*_OXA-72_ as their carbapenem resistance determinant. Two were IC6 isolates, Pasteur ST78, and in possession of *bla*_CTX-M-115_, similar to V86042_BER_HH and V58143_5_KOB in this study [42]. Taken together, these data suggest a circulating IC6 clone in Ukraine and Russia. Interestingly, two isolates had the class A beta-lactamase bla_GES-12_, one of which was the sole carbapenem resistance determinant. The isolates, from Koblenz and Berlin, were closely related by cgMLST (four alleles different), but differed in their resistome, with one isolate having *bla*_OXA-23_ and resistance determinants to chloramphenicol, tetracycline, and some aminoglycosides that were not present in the other. Again, this is suggestive that a mobile genetic element such as a plasmid is present in one isolate. Biochemically, *bla*_GES-12_ encodes an ESBL (extended spectrum beta-lactamase) with little carbapenemase activity [43], however it has been shown to confer carbapenem resistance to *A. baumannii* transformants, particularly against meropenem [43].

Despite two isolates having the fluoroquinolone/aminoglycoside resistance determinant *aac(6′)Ib-cr-like*, fluoroquinolone resistance was associated in all the isolates with the classic amino acid substitutions in Ser83-Leu and Ser80-Leu (GyrA/ParC, respectively) [44].

## 4. Materials and Methods

### 4.1. Patient Isolates

From patients who were war-injured during the Eastern Ukranian conflict and evacuated to Germany for medical treatment at 4 Bundeswehr Hospitals in Berlin, Hamburg, Koblenz and Ulm in 2014 and 2015, a total of 32 *A. baumannii* were isolated and subjected to preliminary typing by “DiversiLab” (BioMérieux, Marcy l’Étoile, France) rep-PCR as recently described, and as openly accessible for readers with interest in procedural details [34]. From 4 major rep-PCR-defined clonal clusters and several outstanders, altogether 8 isolates were randomly chosen for sequence-based further assessment. In detail, the isolates V86041-BER-HH (rep-PCR-cluster 1), V86039-BER-HH (rep-PCR-cluster 2), V66728-3-KOB (rep-PCR-cluster 3), V60248-1-KOB (rep-PCR-cluster 4), V77717-2-KOB (outlier close to rep-PCR-cluster 3), V86042-BER-HH (outlier close to rep-PCR-cluster 4), V58143-5-KOB (singleton) and V86030-BER-HH (singleton) were included in the study. The isolate codes consist of the laboratory number and the Bundeswehr Hospitals where the samples were collected (KOB = Koblenz, BER-HH = Berlin or Hamburg, as samples from both Bundeswehr Hospitals were assessed by the same laboratory). All observed, potential clonal differences as indicated by rep-PCR [34] were covered by this choice.

### 4.2. DNA Extraction and Whole Genome Sequencing

The genomic DNA of all isolates was extracted using the MagAttract HMW DNA Kit (Qiagen, Hilden, Germany) following the manufacturer’s instructions, and was used for whole genome sequencing (WGS). Sequencing libraries were prepared using the Nextera XT library prep kit (Illumina GmbH, Munich, Germany) for a 250 bp paired-end sequencing run on an Illumina MiSeq sequencer. The obtained reads were assembled de novo with use of the Velvet assembler integrated in the Ridom SeqSphere+ v.7.0.4. The raw sequencing reads generated in this project were submitted to the European Nucleotide Archive (https://www.ebi.ac.uk/ena/) under the Accession numbers ERR4436804 (V58143-5-KOB), ERR4437598 (V60248-1-KOB), ERR4437599 (V66728-3-KOB), ERR4437600 (V77717-2-KOB), ERR4437601 (V86030-BER-HH), ERR4437831 (V86039-BER-HH), ERR4438930 (V86041-BER-HH) and ERR4438931 (V86042-BER-HH).

### 4.3. Molecular Epidemiology and Determination of Antibiotic Resistance Genes

Sequence types (STs) according to the Oxford and the Pasteur 7-loci MLST schemes [39,45,46,47] were derived from the genome assemblies of all isolates using the pubMLST website (https://pubmlst.org/abaumannii/).

The isolates were further investigated by applying a validated core genome multi-locus sequence typing (cgMLST) scheme, as described recently [37], using the Ridom SeqSphere^+^ v. 7.0.4 software (Ridom GmbH, Münster, Germany). A minimum spanning tree including 2390 target alleles was generated to visualize their clonal relatedness using the same software. The isolates were also compared to our in-house library to investigate their relationship to the established international clones. The software Resfinder [48,49] was applied to identify resistance genes as described, and the beta-lactamase database was used to identify the *bla*_ADC_ variants (http://www.bldb.eu/).

### 4.4. Ethical Clearance

Ethical clearance for the molecular typing of the *Acinetobacter* strains isolated from the Ukrainian patients was obtained from the ethics committee of the medical association of Hamburg (WF-029/15) in line with National German laws, without the need for informed consent.

## 5. Conclusions

In conclusion, this study provided epidemiological information on carbapenem-resistant *A. baumannii* isolates obtained from patients from the Eastern Ukrainian crisis zone. Epidemiology on the distribution and spread of antimicrobial resistance in international war and crisis zones is a neglected field of research, as surveillance assessments in respective regions are difficult to perform. An assessment of eight randomly selected strains cannot replace broader surveillance efforts. However, considering the scarcely available information on the local epidemiology of carbapenemases in the Ukraine [31,32,33] and, in contrast, the frequent detection of carbapenem resistance in clinical *A. baumannii* isolates from severely infected Ukrainian patients [28,29], these data may at least provide a piece in the puzzle of global antimicrobial resistance. Future studies on the local epidemiology of carbapenem resistance in Ukrainian *A. baumannii* isolates are necessary to further amend this preliminary information.

## Figures and Tables

**Table 1:** Analysis of antimicrobial resistance determinants, ordered by strain, MLST type, and the international clonal lineage of the assessed *A. baumannii* isolates.

Sample (rep-PCR Cluster)	MLST		Clonal Lineage	Antibiotic Resistance Determinants							
	STox	STpas		Sulphonamide	Phenicol	Beta-Lactam	Aminoglycoside	Macrolide	Tetracycline	Trimethoprim	Fluoroquinolone and Aminoglycoside
V86041-BER-HH (rep-PCR 1)	1102	2	IC2			*bla*_ADC-11_ *, bla*_OXA-23_ *,* *bla*_OXA-66_	*aac(3)-Ia-like, aadA1-like, aph(3′)-Ic, aph(3′)-VIa-like*				
V86039-BER-HH (rep-PCR 2)	231	1	IC1	*sul1*	*catA1-like, cmlA1-like*	*bla*_ADC-158_ *, bla*_GES-12_ *,* *bla*_OXA-23_ *, bla*_OXA-69_	*aac(3)-Ia-like, aadA1, aadA2, aadB, aph(3′)-VIa-like, strA-like, strB-like*		*tet(A)*	*dfrA7*	*aac(6′)Ib-cr-like*
V66728-3-KOB (rep-PCR 3)	231	1	IC1	*sul1*	*cmlA1-like*	*bla*_ADC-158_ *, bla*_GES-12_ *,* *bla*_OXA-69_	*aadA2, aadB, aph(3′)-VIa, strA-like, strB-like*			*dfrA7*	*aac(6′)Ib-cr-like*
V77717-2-KOB (rep-PCR 3 outlier)	440	25	IC7	*sul2*		*bla*_ADC-26-like_ *, bla*_OXA-64_	*aadB-like, aph(3′)-Ic, strA, strB-like*				
V60248_1_KOB (rep-PCR 4)	944	78	IC6	*sul1*		*bla*_ADC-152_ *, bla*_OXA-90_ *,* *bla*_OXA-72_	*aadA5, armA*	*mph(E)*			
V86042_BER_HH (rep-PCR 4 outlier)	944	78	IC6	*sul1, sul2-like*	*catA1-like, floR-like*	*bla*_ADC-152_ *, bla*_CARB-16_ *, bla*_CTX-M-115_ *, bla*_OXA-90_ *, bla*_OXA-72_	*aadA5, armA*	*mph(E)*			
V58143_5_KOB (singleton)	2144	78	IC6	*sul2-like*	*catA1-like, floR-like*	*bla*_ADC-107_ *, bla*_CTX-M-115_ *, bla*_OXA-90_ *, bla*_OXA-72_					
V86030_BER_HH (singleton)	690	25	IC7	*sul2*		*bla*_ADC-26-like_ *, bla_OXA-23_ *, bla*_OXA-64_	*aph(3′)-Ic, aph(3′)-VIa-like, strA-like, strB*				

* The enzyme ADC-26-like has two substitutions compared to ADC-26, and the amino-acid sequence was as follows: >ADC-26-like MRFKKISCLLLSPLFIFSTSIYAGNTPKDQEIKKLVDQNFKPLLEKYDVPGMAVGVIQNNKKYEMYYGLQSVQDKKAVNSSTIFELGSVSKLFTATAGGYAKNKGKISFDDTPGKYWKVLKNTPIDQVNLLQLATYTSGNLALQFPDEVQTDQQVLTFFKDWKPKNPIGEYRQYSNPSIGLFGKVVALSMNKPFDQVLEKTIFPALGLKHSYVNVPKTQMQNYAFGYNQENQPIRVNPGPLDAPAYGVKSTLPDMLSFIHANLNPQKYPADIQRAINETHQGFYQVNTMYQALGWEEFSYPATLQTLLDSNSEQIVMKPNKVTAISKEPSVKMYHKTGSTTGFGTYVVFIPKENIGLVMLTNKRIPNEERIKAAYAVLNAIKK.
